# Barriers, motivators and facilitators related to prenatal care utilization among inner-city women in Winnipeg, Canada: a case–control study

**DOI:** 10.1186/1471-2393-14-227

**Published:** 2014-07-15

**Authors:** Maureen I Heaman, Michael Moffatt, Lawrence Elliott, Wendy Sword, Michael E Helewa, Heather Morris, Patricia Gregory, Lynda Tjaden, Catherine Cook

**Affiliations:** 1 College of Nursing, Faculty of Health Sciences, University of Manitoba, 89 Curry Place, Winnipeg, MB R3T 2N2, Canada; 2 Department of Community Health Sciences, College of Medicine, Faculty of Health Sciences, University of Manitoba, Winnipeg, MB R3E 0W3, Canada; 3 Department of Obstetrics, Gynecology & Reproductive Sciences, College of Medicine, Faculty of Health Sciences, University of Manitoba, Winnipeg, MB R3E 0L8, Canada; 4 Department of Pediatrics and Child Health, College of Medicine, Faculty of Health Sciences, University of Manitoba, Winnipeg, MB R3A 1S1, Canada; 5 Department of Medical Microbiology, College of Medicine, Faculty of Health Sciences, University of Manitoba, Winnipeg, MB R3E 0J9, Canada; 6 School of Nursing and Department of Clinical Epidemiology and Biostatistics, Faculty of Health Sciences, McMaster University, Hamilton, ON L8N 3Z5, Canada; 7 Faculty of Nursing, University of Alberta, Edmonton, AB T5G1C9, Canada; 8 Women’s Health Program, Winnipeg Regional Health Authority, Winnipeg, MB R3E 0L8, Canada; 9 Public Health, Winnipeg Regional Health Authority, Winnipeg, MB R3A 0X7, Canada; 10 Population and Aboriginal Health, Winnipeg Regional Health Authority, Winnipeg, MB R3B 1E2, Canada

**Keywords:** Pregnancy, Prenatal care, Access to care, Barriers, Motivators, Facilitators, Inner-city women, Health care utilization

## Abstract

**Background:**

The reasons why women do not obtain prenatal care even when it is available and accessible are complex. Despite Canada’s universally funded health care system, use of prenatal care varies widely across neighborhoods in Winnipeg, Manitoba, with the highest rates of inadequate prenatal care found in eight inner-city neighborhoods. The purpose of this study was to identify barriers, motivators and facilitators related to use of prenatal care among women living in these inner-city neighborhoods.

**Methods:**

We conducted a case–control study with 202 cases (inadequate prenatal care) and 406 controls (adequate prenatal care), frequency matched 1:2 by neighborhood. Women were recruited during their postpartum hospital stay, and were interviewed using a structured questionnaire. Stratified analyses of barriers and motivators associated with inadequate prenatal care were conducted, and the Mantel-Haenszel common odds ratio (OR) was reported when the results were homogeneous across neighborhoods. Chi square analysis was used to test for differences in proportions of cases and controls reporting facilitators that would have helped them get more prenatal care.

**Results:**

Of the 39 barriers assessed, 35 significantly increased the odds of inadequate prenatal care for inner-city women. Psychosocial issues that increased the likelihood of inadequate prenatal care included being under stress, having family problems, feeling depressed, “not thinking straight”, and being worried that the baby would be apprehended by the child welfare agency. Structural barriers included not knowing where to get prenatal care, having a long wait to get an appointment, and having problems with child care or transportation. Attitudinal barriers included not planning or knowing about the pregnancy, thinking of having an abortion, and believing they did not need prenatal care. Of the 10 motivators assessed, four had a protective effect, such as the desire to learn how to protect one’s health. Receiving incentives and getting help with transportation and child care would have facilitated women’s attendance at prenatal care visits.

**Conclusions:**

Several psychosocial, attitudinal, economic and structural barriers increased the likelihood of inadequate prenatal care for women living in socioeconomically disadvantaged neighborhoods. Removing barriers to prenatal care and capitalizing on factors that motivate and facilitate women to seek prenatal care despite the challenges of their personal circumstances may help improve use of prenatal care by inner-city women.

## Background

Prenatal care is a widely used preventive health care service, with the potential to improve health outcomes for women and infants. Despite the emphasis placed on the value of this care [1,2], disparities in utilization of prenatal care persist even in countries with universal access to health care. Our previous research found wide variation in the proportion of women receiving inadequate prenatal care in the Canadian province of Manitoba, with rates ranging from 1.1% to 21.5% across the 25 neighborhoods of the capital city of Winnipeg [3]. The eight neighborhoods with the highest rates of inadequate prenatal care were clustered in or near the inner-city, and are characterized by lower average family incomes and higher rates of single parent families, unemployment, Aboriginal people, and recent immigrants compared to suburban areas. An additional file shows these characteristics in more detail (see Additional file 1).

Numerous studies have explored the reasons underlying inadequate prenatal care, using the notion of barriers as perceptions of the potentially negative aspects or costs associated with a particular behavior [4,5]. Looking at health care broadly, Stewart has proposed that social, cultural, psychological and organizational barriers influence the complex, multivariate nature of access to care for economically disadvantaged Canadians [6].

Significant personal barriers to prenatal care include feeling negatively about the pregnancy or considering abortion [7-15]; generally feeling depressed, unwell, tired, or overwhelmed by personal problems [13,16]; and lacking motivation to learn how to protect one’s health or having negative attitudes towards prenatal care [7,8,15,16]. Women commonly identify a lack of transportation to get to clinic appointments, problems with child care, and a long waiting time at appointments as situational barriers to care [9,11,13,16-18]. Other barriers include negative attitudes of health care providers and staff, non-inclusion of male partners in the prenatal experience, and personal fear of medical examination or procedures [17,19]. Having moved recently or not having a regular health care provider before pregnancy are additional barriers [10,20]. In the U.S., lack of health insurance or being a Medicaid recipient is an additional significant barrier to prenatal care [9,21-24].

In contrast to the extensive literature on barriers, a review by Phillippi found a much smaller body of research on factors that motivate women to access prenatal care [25]. Women who perceive prenatal care as important use the services significantly more than other women [26]. Other important motivators include the mother’s belief that prenatal care will ensure a healthy baby and having someone who encouraged them to seek prenatal care [13]. In a study of low-income women, participants identified several benefits of prenatal care (learning how the baby is doing, learning better health habits, and learning about labor and delivery) and viewed concern about the baby’s well-being as a motivator for them to seek care [27]. Pregnant adolescents also reported having a healthy baby as a primary motivation for prenatal care [17].

To date, most studies of factors related to use of prenatal care have been conducted in the U.S. Many of these studies focused on African American and Hispanic women, or women receiving Medicaid. Given differences in the health care system and racial/ethnic composition between the U.S. and Canada, the results of these studies are not directly generalizable to a Canadian population, where women receive prenatal care through a universal, publicly funded system and where a considerable proportion of the population is Aboriginal. This research was part of a larger, mixed-methods project with the overall goal of identifying determinants of inadequate PNC among inner-city women living in Winnipeg, Manitoba, Canada. The project consisted of a qualitative descriptive study that involved interviews with pregnant women and prenatal care providers in the inner-city, and a case–control study of postpartum women from which the findings of a multivariable logistic regression analysis to identify demographic, behavioral and psychosocial risk factors for inadequate prenatal care will be reported separately. The specific aims of this component of the case–control study were (1) to compare the proportion of barriers, motivators and facilitators of prenatal care utilization reported by inner-city women who received inadequate prenatal care (cases) and those who received adequate prenatal care (controls), and (2) to measure the strength of association between the exposures (barriers and motivators) and the outcome of inadequate prenatal care. The findings will fill a gap in knowledge related to factors influencing use of prenatal care in Canada, and thereby provide evidence to inform policy and practice at both a local and national level. In addition to learning more about barriers, understanding the factors that can motivate or facilitate a woman to seek prenatal care despite difficult personal circumstances may contribute to building effective, strength-based interventions to improve utilization of prenatal care.

## Methods

### Setting, case definition and subject selection

Women were recruited for this case–control study during their postpartum stay at either of the two hospitals providing maternity care in Winnipeg, Manitoba. Since 2005, all deliveries in the city have taken place at these two hospitals (approximately 10,000–11,000 births per year in total) with the exception of a small number of home births. Winnipeg has a population of 633,745 people, with the highest proportion of Aboriginal people (10%) of all capital cities in Canada [28].

Inadequate prenatal care has been defined in a variety of ways, such as late initiation of prenatal care, having less than a specified number of visits, or as a category using one of the existing prenatal care utilization indices [29-31]. In this study, inadequate prenatal care was defined as (a) having no prenatal care; or (b) prenatal care that began in the third trimester (at 28 weeks gestation or later), irrespective of the total number of prenatal care visits; or (c) prenatal care that began in the first or second trimester but with a low number of visits using the following criteria: women delivering at term or at 34+ weeks with 1–4 visits in total, those delivering at 32–33 weeks with 1–3 visits in total, those delivering at 30–31 weeks with 1–2 visits in total, or those delivering at ≤ 29 weeks with only 1 visit (adapted from the GINDEX [29]). Adequate prenatal care was defined based on the GINDEX [29] as having an initial visit in the first trimester (< = 13 weeks) and having 4 or more visits for women delivering at 22–25 weeks gestation, 5 or more visits at 26–29 weeks, 6 or more visits at 30–31 weeks, 7 or more visits at 32–33 weeks, 8 or more visits at 34–35 weeks, or 9 or more visits at 36+ weeks. A prenatal visit was defined as a visit to a health professional (e.g., physician, midwife or nurse practitioner) where some kind of medical act was performed to take care of the pregnancy; visits intended only to confirm pregnancy were not included (adapted from Delvaux [32]).

Cases were women who had given birth to a live infant, resided in one of the eight Winnipeg neighborhoods with rates of inadequate prenatal care of 5% or higher (as identified in our earlier study [3]), and received inadequate prenatal care. Controls were women who had given birth to a live infant, resided in one of the same eight Winnipeg neighborhoods, and received adequate prenatal care during their pregnancy. To gain a broad perspective, we included women with either a singleton or multiple birth, women of all ages, including adolescents, women whose infants were placed under the care of Child and Family Services (the province’s child welfare agency), and women who spoke English, French, or one of four immigrant languages most common in Winnipeg (Tagalog, Punjabi, Vietnamese, Arabic). We excluded women who were categorized as having intermediate prenatal care using the GINDEX [29], women with a known psychiatric disorder that precluded participation in the interview and women who had an early neonatal death because it would have been inappropriate for ethical reasons to interview these women during the grieving process.

Sample size was estimated using StatCalc from Epi Info, Version 3.3.2, based on the following parameters: one-sided alpha of 5%, power of 80%, minimum detectable odds ratio of 2.0, exposure rate among controls of 8% (estimated from Johnson et al.’s results [8,33]), and a ratio of controls per case of 2:1. This yielded a required sample size of 195 cases and 390 controls, for a total of 585 women.

### Procedure

Approval to conduct the study was received from the University of Manitoba Education/Nursing Research Ethics Board, the St. Boniface General Hospital Research Review Committee, and the Health Sciences Centre Research Impact Committee, as well as the Assembly of Manitoba Chiefs Health Information Research Governance Committee. Research nurses with maternal-child health care experience were hired to recruit women at the two hospitals seven days per week with the exception of statutory holidays. The research nurses screened potential participants for eligibility with the assistance of hospital staff. Postal codes were used to determine if the woman resided in one of the eight eligible neighborhoods. All eligible women who agreed to receive an explanation about the study were approached during their postpartum stay to participate in the study. Potential participants were provided with a written letter of invitation (translated into five languages) and a verbal explanation about the study. Women who agreed to participate in the structured interview then signed a consent form.

The majority of interviews (95%) took place in the hospital setting with the remainder occurring in the home postpartum. Participation in the interview took 46 minutes on average. Interpreters were used to facilitate interviews with women who spoke Tagalog (n = 5), Vietnamese (n = 3) and Arabic (n = 1). Although interpreters were available to assist women who spoke Punjabi or French, this was not requested by any participant. Upon completion of the interview, each woman received a $20 grocery store coupon in compensation for her time. Because rates of inadequate prenatal care varied from 5.0% to 21.5% across the eight neighborhoods included in the study, frequency matching by neighborhood was used. We recruited double the number of controls to cases from each neighborhood. Data collection commenced in January 2007 and ended in January 2010.

The research nurses were trained in structured interview techniques, ethical considerations, and administrative procedures. A pilot test was conducted during which each research nurse conducted two interviews. The research nurses, the project coordinator, and the principal investigator then met to discuss problems encountered and made decisions on a consistent approach to be used. The pilot test was also used to determine if the length of the questionnaire was appropriate. Pilot test results were not included in the final sample.

### Data collection

Most case–control studies rely on a questionnaire as the primary source of exposure data [34]. A structured interview was conducted with each participant, using a standardized questionnaire to reduce error. An interview approach was selected over a self-administered questionnaire because of the role the interviewer could play in guiding the questioning, enhancing respondent participation, answering the respondent’s questions, and clarifying the meaning of responses [35].

The questionnaire consisted of closed-ended questions regarding barriers, motivators, and facilitators related to use of prenatal care. Refer to Additional file 2 for wording of the questions and response options. Barriers (39 items) were defined as “any state, condition or event that made it difficult or prevented a woman from obtaining prenatal care” [8]. Motivators (10 items) were internal or psychological factors that stimulated a woman to seek prenatal care. Facilitators (11 items) were external factors that would have helped a woman get more prenatal care than she did or made it easier to access prenatal care. The majority of items were adapted with permission from a questionnaire developed by Johnson and colleagues to assess determinants of prenatal care utilization in Washington, DC [8,33]. Some of the original items were deleted because they were not relevant to the Canadian context (e.g., no health insurance, no money to pay for prenatal care). Each section of the questionnaire was preceded by a statement and question, and women responded with *yes*, *no*, or *not applicable*.

The questionnaire was also used to collect data on sociodemographic characteristics and the amount, timing, provider and location of prenatal care. Several of the sociodemographic and pregnancy questions were adapted from widely used surveys such as the National Population Health Survey (Statistics Canada) [36] and the Pregnancy Risk Assessment Monitoring System (Centers for Disease Control) [37]. This enhanced the quality of the questionnaire because these questions have been devised and tested by survey experts. Content validation of the questionnaire was performed by having it reviewed by several experts and through pilot testing with potential respondents.

### Data analysis

Data analysis was performed using SPSS Statistics for Windows, Version 19.0. A statistician from the Manitoba Centre for Nursing and Health Research assisted with the analyses. Initially, data were summarized using descriptive statistics; frequencies were calculated for categorical variables, and means and distribution were calculated for continuous variables. The chi-square test was used to test for differences in proportions for demographic and prenatal care variables and for facilitators among cases and controls. The independent t-test was used to test for differences in means for continuous demographic and prenatal care variables. We examined differences in the frequency of cases and controls reporting exposure to each of the barriers and motivators, and calculated the ratio of the odds of exposure in the case group to the odds of exposure in the control group as a measure of the strength of the association between an exposure and the outcome of inadequate prenatal care. Because cases were frequency matched to controls 1:2 by neighborhood, a stratified analysis was used to evaluate and describe effect-measure homogeneity between neighborhood strata. When the Breslow-Day test for homogeneity of odds ratios was not rejected, the Mantel-Haenszel common odds ratio (OR) and 95% confidence interval (CI) were calculated to provide a weighted average of the subgroup-specific OR.

## Results

### Sample characteristics

The final sample consisted of 406 controls (adequate prenatal care) and 202 cases (inadequate prenatal care). Demographic, prenatal care and pregnancy-related characteristics of the subjects are described in Tables 1 and 2. Cases were more likely to be younger, multiparous, single/divorced/separated, self-identify as Aboriginal, not have a paid job, and have lower education and family income compared to controls. Of note, 77.7% of cases versus 31.3% of controls reported a family income of < $20,000 per year, and 85.1% of cases versus 33.2% of controls self-identified as Aboriginal. In keeping with the inclusion criteria for each group, cases had their first prenatal visit at a mean of 21.1 weeks (SD 9.2) compared to 9.0 weeks (SD 2.8) for controls. On average, cases had only 3.3 prenatal visits (SD 1.5) compared to 12.1 visits (SD 2.5) among controls. Thirty cases reported having no prenatal care. Cases were much further advanced in their pregnancy before knowing they were pregnant: 12 weeks (SD 8.4) on average, while controls were 5.9 weeks (SD 3.3).

**Table 1:** Background characteristics (categorical variables) of women with inadequate versus adequate prenatal care (PNC), Winnipeg, MB, 2007–2010

**Characteristic**^**1**^	**Cases (inadequate PNC)**	**Controls (adequate PNC)**	**Chi square**	**p value**
	**n = 202**	**n = 406**		
	**n (%)**	**n (%)**		
**Marital status**				
Married	12 (6)	169 (41.8)	94.94	<.001
Common-law	68 (33.8)	123 (30.4)		
Single/divorced/separated	121 (60.2)	112 (27.7)		
**Total family income**				
< = $19,999	143 (77.7)	118 (31.3)	112.66	<.001
$20,000-$39,999	27 (14.7)	106 (28.1)		
$40,000-$59,999	9 (4.9)	66 (17.5)		
> = $60,000	5 (2.7)	87 (23.1)		
**Years of education completed**				
< = 8 years	32 (15.9)	13 (3.2)	129.87	<.001
9-11 years	119 (59.2)	106 (26.4)		
12 years	33 (16.4)	89 (22.2)		
> = 13 years	17 (8.5)	193 (48.1)		
**Racial background**				
Aboriginal	171 (85.1)	134 (33.2)	147.44	<.001
Black	0 (0)	14 (3.5)		
White	24 (11.9)	162 (40.1)		
Filipino	1 (0.5)	49 (12.1)		
Other	5 (2.5)	45 (11.1)		
**Parity (prior to birth)**				
0	43 (21.3)	201 (49.5)	99.06	<.001
1	48 (23.8)	121 (29.8)		
2	30 (14.9)	48 (11.8)		
3	36 (17.8)	20 (4.9)		
> = 4	45 (22.3)	16 (3.9)		
**Weeks pregnant at first PNC visit**^**2**^				
< = 13 weeks	50 (30.1)	406 (100)	355.88	<.001
14-27 weeks	71 (42.8)	0 (0)		
> = 28 weeks	45 (27.1)	0 (0)		
**Number of PNC visits**^**2**^				
< = 4 visits	156 (91.2)	0 (0)	519.35	<.001
5-9 visits	13 (7.6)	44 (10.8)		
10-14 visits	2 (1.2)	295 (72.7)		
> = 15 visits	0 (0)	67 (16.5)		
**Had a paid job during pregnancy**				
Yes	31 (15.4)	262 (64.9)	131.30	<.001
No	170 (84.6)	142 (35.1)		

^1^7.7% of data were missing for the income variable and ≤ 1.0% of data were missing for all other characteristics. Missing data were excluded from analyses.

^2^Excludes women with no prenatal care visits (n = 30).

**Table 2:** Background characteristics (continuous variables) of women with inadequate versus adequate prenatal care (PNC), Winnipeg, MB, 2007–2010

**Characteristic**	**Cases (inadequate PNC)**	**Controls (adequate PNC)**	**t-test**	**p value**
	**n = 202**	**n = 406**		
	**Mean ( *SD *)**	**Mean ( *SD *)**		
Maternal age (years)	24.6 (5.8)	26.8 (6.2)	-4.23	<.001
Education (years)	10.2 (1.8)	13.0 (3.1)	-11.95	<.001
Parity	3.2 (2.0)	1.9 (1.1)	10.70	<.001
Weeks pregnant when sure pregnant	12.0 (8.4)	5.9 (3.3)	9.79	<.001
Number of PNC visits^1^	3.3 (1.5)	12.1 (2.5)	-52.34	<.001
Weeks pregnant at first PNC visit^1^	21.1 (9.2)	9.0 (2.8)	16.70	<.001

^1^Calculation of mean excludes 30 cases who had no prenatal care.

Most barriers and motivators were homogeneous across neighborhoods, therefore the Mantel-Haenszel common ORs are reported (Tables 3 and 4). Because of the “not applicable” option, not all women responded “yes” or “no” to each of the questions. For example, the questions on reasons for getting prenatal care were not applicable to women who had no prenatal care; the questions about child care were not applicable to women who did not have other children; and the questions about work schedule or time off work were not applicable to women who were unemployed or on social assistance. One of the cases did not realize she was pregnant until she gave birth in the Emergency Room; therefore she did not respond to any of the questions on barriers with the exception of a “yes” response to the item, “you did not know you were pregnant.” Therefore the number of respondents who replied either “yes” or “no” to each item is reported in Tables 3 and 4 to provide a denominator for the percentages; the “not applicable” responses were excluded from the analyses.

**Table 3:** Proportion of cases and controls reporting barriers to prenatal care (PNC) utilization and the association with inadequate prenatal care (Mantel-Haenszel odds ratio and 95% confidence interval [CI]) among inner-city women, Winnipeg, MB, 2007–2010

**Barriers**	**Cases (inadequate PNC)**	**Controls (adequate PNC)**	**Mantel-Haenszel odds ratio**	**95% CI**
	**n = 202**	**n = 406**		
	**n (%)**	**n (%)**		
	**Total n**^**1 **^**(denominator)**	**Total n**^**1 **^**(denominator)**		
**Negative attitudes toward pregnancy**				
Unplanned pregnancy	79 (39.5)	32 (7.9)	7.54	4.77 – 11.93
	n = 200	n = 406		
Considering abortion	53 (26.4)	7 (1.7)	20.03	8.83 – 45.40
	n = 201	n = 406		
Unaware of pregnancy	49 (24.4)	8 (2.0)	16.07	7.48 – 34.54
	n = 201	n = 405		
Unhappy about pregnancy	32 (15.9)	8 (2.0)	9.41	4.25 – 20.87
	n = 201	n = 406		
Wanted to hide pregnancy	37 (18.4)	5 (1.2)	16.90	6.72 – 42.51
	n = 201	n = 406		
**Negative attitudes toward prenatal care**				
Go to emergency room or OB triage unit when problems occur	72 (35.8)	30 (7.4)	7.09	4.41 – 11.40
	n = 201	n = 406		
Receive advice about pregnancy from family/friends	90 (44.8)	37 (9.1)	8.26	5.30 – 12.87
	n = 201	n = 406		
Can take care of self during pregnancy	103 (51.2)	25 (6.2)	15.79	9.68 – 25.75
	n = 201	n = 406		
Did not think you needed PNC	50 (24.9)	3 (0.7)	45.65	13.93 – 149.58
	n = 201	n = 406		
Afraid of/do not like medical tests and exams	51 (25.4)	8 (2.0)	17.30	7.93 – 37.76
	n = 201	n = 406		
Do not like needles or medications	56 (27.9)	15 (3.7)	10.39	5.65 – 19.09
	n = 201	n = 406		
Dissatisfied with care received	32 (16.0)	13 (3.2)	5.79	2.96 – 11.32
	n = 200	n = 406		
Do not like health care workers	14 (7.0)	5 (1.2)	6.28	2.18 – 18.07
	n = 201	n = 406		
Forgot appointment	102 (50.7)	63 (15.5)	5.97	4.00 – 8.89
	n = 201	n = 406		
**Psychosocial stress**				
Under stress	110 (54.7)	38 (9.4)	11.55	7.47 – 17.84
	n = 201	n = 406		
Depressed	65 (32.3)	18 (4.4)	10.15	5.81 – 17.73
	n = 201	n = 406		
Personal problems	79 (39.3)	23 (5.7)	10.85	6.52 – 18.06
	n = 201	n = 406		
Not thinking straight	55 (27.4)	9 (2.2)	17.10	8.16 – 35.82
	n = 201	n = 406		
Did not feel well	97 (48.3)	69 (17.0)	4.67	3.18 – 6.86
	n = 201	n = 406		
Family problems	71 (35.3)	12 (3.0)	18.01	9.41 – 34.45
	n = 201	n = 406		
Did not feel good about self	48 (23.9)	13 (3.2)	9.57	5.03 – 18.19
	n = 201	n = 405		
Problems with husband/boyfriend	40 (19.9)	10 (2.5)	9.23	4.51 – 18.89
	n = 201	n = 396		
Physically abused by husband or boyfriend	11 (5.5)	2 (0.5)	11.44	2.51 – 52.21
	n = 200	n = 396		
Worried about risk of baby being apprehended by Child and Family Services	42 (20.9)	5 (1.2)	20.75	8.11 – 53.07
	n = 201	n = 406		
**Structural**				
Unaware of where to go for PNC	53 (27.0)	32 (8.1)	4.17	2.58 – 6.72
	n = 196	n = 394		
Could not get an appointment	54 (27.8)	29 (7.3)	4.85	2.97 – 7.93
	n = 194	n = 398		
Long wait to get an appointment	78 (40.2)	41 (10.3)	5.77	3.75 – 8.89
	n = 194	n = 398		
Appointment cancelled by clinic	21 (11.1)	26 (6.5)	1.76	0.96 – 3.21
	n = 189	n = 399		
Clinic hours inconvenient	32 (16.8)	32 (8.1)	2.30	1.37 – 3.89
	n = 190	n = 396		
Had to wait too long in waiting room to see provider	65 (35.3)	86 (21.6)	1.99	1.35 – 2.93
	n = 184	n = 398		
Didn’t think you could communicate with staff	21 (11.0)	27 (6.8)	1.68	0.92 – 3.06
	n = 191	n = 396		
Did not like staff attitudes	22 (11.6)	29 (7.3)	1.68	0.94 – 3.00
	n = 189	n = 399		
**Lack of Personal and Economic Resources**				
Child care problems	59 (41.3)	18 (8.3)	7.58	4.21 – 13.66
	n = 143	n = 217		
Transportation problems	92 (48.4)	49 (12.6)	6.44	4.26 – 9.74
	n = 190	n = 390		
Moving a lot	45 (22.4)	10 (2.5)	11.01	5.45 – 22.22
	n = 201	n = 406		
Were or are homeless	14 (7.0)	3 (0.7)	9.93	2.82 – 34.94
	n = 201	n = 406		
Inability to take time off work	6 (9.1)	18 (6.1)	1.64	0.61 – 4.41
	n = 66	n = 293		
**Miscellaneous**				
Did not want to be examined by a man	62 (31.0)	32 (7.9)	5.19	3.25 – 8.28
	n = 200	n = 404		
Fear of crime near the home or clinic	21 (10.4)	16 (3.9)	2.87	1.46 – 5.64
	n = 201	n = 406		

^1^The total n, or denominator for the percentage reported, reflects the number of cases or controls who responded either “yes” or “no” to each item and excludes those who responded “not applicable” or who had no response to that item.

**Table 4:** Proportion of cases and controls reporting motivators of prenatal care (PNC) utilization and the association with inadequate PNC (Mantel-Haenszel odds ratio and 95% confidence interval ([CI]) among inner-city women, Winnipeg, MB, 2007–2010

**Motivators**	**Cases (inadequate PNC)**	**Controls (adequate PNC)**	**Mantel-Haenszel odds ratio**	**95% CI**
	**n = 202**	**n = 406**		
	**n (%)**	**n (%)**		
	**Total n**^**1 **^**(denominator)**	**Total n**^**1 **^**(denominator)**		
Have a healthy baby	164 (96.5)	401 (98.8)	0.33	0.10 – 1.10
	n = 170	n = 406		
Learn better health habits	99 (58.2)	289 (71.2)	0.57	0.39 – 0.82
	n = 170	n = 406		
Learn how to protect your health	95 (55.9)	305 (75.31)	0.41	0.28 – 0.60
	n = 170	n = 405		
Afraid of having problems without care	102 (60.0)	266 (65.5)	0.78	0.54 – 1.14
	n = 170	n = 406		
Talk to someone about pregnancy	102 (59.6)	309 (76.3)	0.46	0.31 – 0.67
	n = 171	n = 405		
Learn about labor and delivery	65 (38.2)	257 (63.6)	0.36	0.25 – 0.52
	n = 170	n = 404		
Encouraged by husband/boyfriend	63 (37.3)	132 (33.0)	1.21	0.83 – 1.76
	n = 169	n = 400		
Encouraged by family members	44 (25.9)	76 (18.9)	1.50	0.98 – 2.30
	n = 170	n = 403		
Encouraged by friends	27 (15.9)	53 (13.2)	1.23	0.74 – 2.04
	n = 170	n = 402		
Encouraged by health care provider or social worker	39 (23.2)	113 (28.8)	0.74	0.49 – 1.13
	n = 168	n = 392		

^1^The total n, or denominator for the percentage reported, reflects the number of cases or controls who responded either “yes” or “no” to each item and excludes those who responded “not applicable” or who had no response to that item.

### Barriers

All of the barriers examined were more frequently reported by cases than controls, and 35 of the 39 barriers assessed were associated with significantly higher odds of inadequate prenatal care (Table 3). All categories of barriers (e.g., attitudinal, psychosocial, structural, and economic) played important roles in under-utilization of prenatal care. The highest odds of inadequate prenatal care were associated with barriers in the categories of “negative attitudes toward pregnancy”, “negative attitudes toward prenatal care”, and “psychosocial issues”.

Negative attitudes toward the pregnancy, primarily due to having an unplanned or unwanted pregnancy, were associated with increased odds of inadequate prenatal care. For example, women who were thinking about having an abortion were 20 times more likely to have inadequate prenatal care. We found high odds ratios for several other attitudinal factors (unaware of being pregnant, OR 16.07; wanted to hide the pregnancy, OR 16.90; unhappy about the pregnancy, OR 9.41).

We also found that a substantial proportion of cases held negative attitudes toward prenatal care itself. Close to 25% of cases did not think they needed prenatal care (versus <1% of controls; OR 45.65) and over one half of cases (versus 6.2% of controls) indicated they could take care of themselves during pregnancy (OR 15.79). Other barriers associated with an increased likelihood of inadequate prenatal care were being afraid of medical tests and exams (OR 17.30), using the emergency room or obstetrical triage unit if problems occur (OR 7.09), and receiving advice about pregnancy from family and friends (OR 8.26). Being dissatisfied with their care, not liking the health care workers, and forgetting the appointment were associated with lower, although still significant, odds of inadequate prenatal care.

Psychosocial issues played a large role in the use of prenatal care by cases compared to controls. Almost one-third of cases reported being depressed during their pregnancy (versus 4.4% of controls; OR 10.15), and more than half reported being under stress (versus 9.4% of controls, OR 11.55). Psychosocial issues associated with the highest likelihood of inadequate prenatal care were not thinking straight (OR 17.10), family problems (OR 18.01), and being worried about the risk of the baby being apprehended by the child welfare agency (OR 20.75).

Cases also consistently identified structural factors as barriers to obtaining prenatal care more often than controls did, although these factors had generally smaller odds ratios than we found for other types of barriers. The most common concerns surrounded challenges with the appointment itself (such as long wait to get an appointment, OR 5.77; could not get an appointment, OR 4.85) or being unaware of where to go for prenatal care (OR 4.17). Structural barriers related to the attitudes or communication abilities of staff were reported the least frequently, and were not significantly associated with an increased likelihood of inadequate prenatal care.

Other barriers were related to a lack of personal and economic resources. Problems with transportation were reported by 48.4% of cases compared to 12.6% of controls (OR 6.44). Child care problems also significantly increased the odds of inadequate prenatal care (OR 6.44). Homelessness and moving a lot were more frequently reported as barriers to prenatal care by the cases than the controls, and were associated with a significantly higher likelihood of inadequate prenatal care. Lastly, not wanting to be examined by a man was associated with a five-fold greater likelihood of inadequate prenatal care.

### Motivators

Four motivators, including three that relate to prenatal care as an opportunity to obtain information, significantly reduced the odds of inadequate prenatal care, thereby serving as protective factors (Table 4). Controls were more likely than cases to be motivated to seek prenatal care to learn about better health habits, how to protect their own health, and labor and delivery. They were also more likely to be motivated by the value of talking to someone about the pregnancy. However, other motivators did not significantly decrease the odds of inadequate prenatal care, such as being encouraged by friends, family members, or a partner to attend care. Interestingly, similar proportions of both cases (96.5%) and controls (98.8%) reported being motivated to obtain prenatal care because of their desire to have a healthy baby.

### Facilitators

A significantly higher proportion of cases than controls responded that 10 out of the 11 facilitators would make “a lot” or “some” difference in helping them get more prenatal care than they did (Table 5). The most frequently identified facilitators were getting rides to the clinic, getting child care assistance, having a home visitor, and getting a call to follow-up on missed appointments; over 40% of cases responded these would make “a lot” of difference. Other facilitators were convenient clinic hours, staff being easy to understand, having financial support, having emotional support, and getting incentives. The facilitator, “the staff were from the same country as you”, did not differ significantly between cases and controls.

**Table 5:** Differences in proportion of cases and controls reporting facilitators that would help them get more prenatal care (PNC), Winnipeg, MB, 2007-2010

**Variable: How much of a difference would it make if…..**	**Degree of difference**	**Cases (inadequate PNC)**	**Controls (adequate PNC)**	**Chi-square**	**p value**
		**n = 202**	**n = 406**		
		**n (%)**	**n (%)**		
You got help with completing forms	A lot	40 (21.1)	42 (12.8)	22.85	<.0001
	Some	45 (29.4)	61 (17.6)		
	A little	29 (10.0)	41 (11.8)		
	None	67 (37.0)	202 (58.4)		
You got incentives such as gifts or money	A lot	45 (22.5)	56 (14.4)	16.73	.001
	Some	35 (17.5)	50 (12.8)		
	A little	32 (16.0)	44 (11.3)		
	None	88 (44.0)	240 (61.5)		
You got rides to the clinic	A lot	111 (56.9)	126 (33.5)	35.74	<.0001
	Some	27 (13.8)	52 (13.8)		
	A little	21 (10.8)	47 (12.5)		
	None	36 (18.5)	151 (40.2)		
You got child care assistance^1^	A lot	67 (49.6)	57 (30.5)	18.70	<.0001
	Some	20 (14.8)	18 (9.6)		
	A little	11 (8.1)	23 (12.3)		
	None	37 (27.4)	89 (47.6)		
You had a home visitor	A lot	82 (45.1)	67 (20.7)	47.61	<.0001
	Some	38 (20.9)	50 (15.4)		
	A little	17 (9.3)	35 (10.8)		
	None	45 (24.7)	172 (53.1)		
The clinic had hours that were convenient for you	A lot	68 (35.4)	119 (32.7)	12.14	.007
	Some	39 (20.3)	54 (14.8)		
	A little	28 (14.6)	33 (9.1)		
	None	57 (29.7)	158 (43.4)		
You got a call to follow up on missed appointments	A lot	85 (44.0)	85 (28.7)	24.99	<.0001
	Some	45 (23.3)	48 (16.2)		
	A little	18 (9.3)	34 (11.5)		
	None	45 (23.3)	129 (43.6)		
The staff were easy to understand	A lot	67 (36.8)	138 (44.4)	12.14	.007
	Some	39 (21.4)	33 (10.6)		
	A little	19 (10.4)	22 (7.1)		
	None	57 (31.3)	118 (37.9)		
The staff were from the same country as you	A lot	27 (16.8)	52 (17.3)	1.34	.720
	Some	13 (8.1)	16 (5.3)		
	A little	9 (5.6)	17 (5.7)		
	None	112 (69.6)	215 (71.7)		
You had financial support	A lot	65 (33.0)	88 (23.1)	16.17	.001
	Some	41 (20.8)	56 (14.7)		
	A little	22 (11.2)	38 (10.0)		
	None	69 (35.0)	199 (52.2)		
You had emotional support	A lot	69 934.8)	114 (29.5)	8.34	.040
	Some	42 (21.2)	58 (15.0)		
	A little	21 (10.6)	42 (10.9)		
	None	66 (33.3)	173 (29.6)		

^**1**^Respondents were women with other children, n = 322.

## Discussion

### Summary of key findings

This study adds to our limited knowledge of factors that contribute to disparities in prenatal care utilization in Canada. It demonstrated that, among women living in inner-city neighborhoods in Winnipeg, cases and controls differed significantly in the barriers and motivators that affected their ability to obtain adequate prenatal care. Psychosocial barriers that significantly increased the likelihood or odds of inadequate prenatal care included being under stress, having family problems, feeling depressed, “not thinking straight”, and being worried that the baby would be apprehended by the child welfare agency. Structural barriers included not knowing where to get prenatal care and having a long wait to get an appointment, while barriers related to lack of personal and economic resources included having problems with transportation or child care, moving a lot, and being homeless. Negative attitudes toward the pregnancy (such as not planning or knowing about the pregnancy, or thinking of having an abortion) and negative attitudes toward prenatal care (such as believing they did not need prenatal care and that they could take care of themselves during pregnancy, or being afraid of medical tests and exams) significantly increased the odds of inadequate prenatal care. Four motivators decreased the odds of inadequate prenatal care and thereby had a protective effect, such as the desire to learn how to protect one’s health. Cases were significantly more likely than controls to indicate that facilitators such as getting assistance with transportation and child care would have helped them get more prenatal care.

### What the findings mean in the context of literature

The demographic characteristics of inner-city women who received inadequate prenatal care are consistent with those described in two recent reviews [25,38]. One noteworthy finding of our study was that women with inadequate prenatal care were significantly more likely to self-identify themselves as Aboriginal (85.1% of cases versus 33.2% of controls). This finding is consistent with our previous findings of higher rates of inadequate care among Aboriginal women at an individual level [39], as well as in neighborhoods with a higher proportion of Aboriginal people [3], emphasizing the need for culturally safe maternal health care [40]. Although Aboriginal people are more likely than other cultural groups in Canada to live with higher unemployment rates and less education [41], poverty alone is unlikely to explain the disparity in prenatal care utilization. In a U.S. study of barriers to prenatal care, Lia-Hoagberg et al. [13] concluded that “poverty and its associated factors appears to exert a greater influence than race on prenatal care participation among low-income women”, but they also noted that participants of American Indian descent were significantly more likely to miss prenatal care appointments due to personal problems when compared to white or African American women. In Canada, the experience of colonization and, more specifically, cultural oppression through the residential school system are widely acknowledged to have contributed to intergenerational social problems among many First Nations families and to influence current relationships of Aboriginal peoples with the mainstream health care system [40,42]. In addition, Aboriginal women tend to have higher fertility rates [41], and higher parity has been identified as a risk factor for inadequate prenatal care. This may be because women see less value in prenatal care if past pregnancies have gone well, or their personal resources may be limited due to the presence of other young children. Finally, it has been proposed that, within Aboriginal culture, pregnancy is considered a normal state of health, rather than an illness, with some individuals viewing prenatal care as an unnecessary medical intervention [43,44].

### Motivators

The most common motivator for both cases and controls—the belief that prenatal care would help them have a healthy baby—is a finding consistent with results of other studies where women reported this belief as a motivator, [8,13] or described learning about the health of the baby as a benefit of prenatal care [27]. In both Johnson et al.’s studies [8,33] and our study, over 95% of women reported that having a healthy baby was a motivator. Similar to Lia-Hoagberg et al. [13], a relatively small proportion of inner-city women in our study were motivated by the encouragement of others (e.g., husband/boyfriend, family members, friends, health care providers), and the odds ratios for these motivators were not significant, suggesting that women’s decision-making about care was largely an independent process. This finding may also be related to the fact that the majority of cases in this study were single and a substantial proportion experienced family and partner relationship problems. Other studies have reported that the partners of low-income women can be either a positive or negative influence on prenatal care utilization [45]. We found a significant protective effect for four motivators. A higher proportion of controls than cases reported being motivated to seek prenatal care to learn better health habits, learn how to protect their own health, have someone to talk to about their pregnancy, and learn about labor and delivery. This might be explained by the lower mean parity of women in the control group (1.1 versus 2.0 for cases), making them less familiar with pregnancy and childbirth.

### Barriers

Across all six categories of barriers to prenatal care explored in this study, 35 of the 39 specific issues examined were significantly associated with an increased likelihood of inadequate prenatal care. This strongly suggests that inadequate utilization of prenatal care is a multidimensional problem that is likely to be effectively addressed only through complex interventions. In addition, the proportion of women with inadequate prenatal care in our study who reported psychosocial, attitudinal, economic and structural barriers using a similar questionnaire was much higher than in Johnson et al.’s study of 246 African American women in Washington, DC [33] (e.g., unplanned pregnancy, 39.5% in our study vs. 28.5% in Johnson’s study; under stress, 54.7% vs. 27.8%; personal problems, 39.3% vs. 27.8%; can take care of self, 51.2% vs. 18.8%; denied need for prenatal care, 24.9% vs. 10.4%; transportation problems, 48.4% vs. 22.2%). These results underscore the extent of the issues experienced by inner-city women in Winnipeg, although differences in sample size, sample characteristics and use of different prenatal care utilization indices to measure inadequate prenatal care may account for some of the discrepancies in findings.

Issues related to having an unplanned or unwanted pregnancy significantly increased the odds of inadequate prenatal care for women in this study. In their meta-synthesis, Downe et al. highlighted the distress associated with unplanned pregnancy and noted that it often led women to deny or delay identifying the pregnancy, feel unprepared for the change in their lives, consider abortion, and be stigmatized by peers [45]. We found that more than one-quarter of cases identified thinking about having an abortion and nearly 20% wanted to hide the pregnancy; these were significant barriers that caused women to delay starting prenatal care or not go for care. This suggests that improving family planning services to minimize unplanned pregnancies may help increase utilization of prenatal care. In addition, these services should incorporate education about the value of prenatal care to familiarize girls and women with the reasons for prenatal care before they get pregnant.

Negative attitudes about prenatal care were prominent barriers and were largely related to women feeling they could address their care needs on their own. While such attitudes are deemed negative in that they impede women from obtaining appropriate care, they likely reflect the protective mechanisms that disadvantaged women develop in response to their challenging lives. Indeed, models of prenatal care that incorporate self-care (e.g., group prenatal care or Centering Pregnancy programs) have been found to promote regular prenatal care attendance, increase satisfaction with care, and increase prenatal knowledge, particularly among disadvantaged women [46-49].

Other studies have identified characteristics of health care providers (insensitivity [50,51], lack of cultural sensitivity [25], and judgmental attitudes [19]) as barriers to seeking or continuing prenatal care among low-income women, whereas friendliness [19], acceptance [19], answering questions [45], and not rushing during the appointment [45] have been shown to facilitate the use of care. In our study, two barriers involving prenatal care providers significantly increased the likelihood of inadequate prenatal care (not liking the health care workers and dissatisfaction with the care they received), whereas two other barriers were not significant (did not like staff attitudes; did not think they could communicate with staff).

The importance of structural barriers related to clinic hours and appointment wait times found in our study is consistent with a number of others that have observed these to be primary issues [25]. While most women would find inconvenient clinic hours and long wait times unacceptable, for some these issues factor more heavily in the decision to forgo prenatal care. It may be that underlying issues in some women’s lives, such as a chaotic lifestyle [45] characterized by crises and disorganization, make inconvenient hours and long waits for prenatal care a lower priority than other challenges they face. For example, Kalmuss et al. reported that one of the top two barriers to seeking prenatal care among low-income women was “needing time and energy to deal with other problems” [52].

Psychosocial factors, such as depression [13,52,53], psychosocial/emotional problems [33,54] and pregnancy-related stress [54] have been identified as barriers to prenatal care use among adolescents, socioeconomically disadvantaged women, and low-income African American women. Some research has found these factors to figure as prominently as structural barriers [8], and in our study, most of the psychosocial issues were associated with higher odds of inadequate prenatal care than the structural barriers. In addition to stress and depression, our study also showed personal and family problems and being physically abused by their husband/boyfriend prevented women from obtaining adequate prenatal care. In a sample with similar characteristics, women with inadequate prenatal care were more likely to report personal and family problems which, in some cases, created high levels of stress, for example when they involved partner abuse or “not getting along well” or when a partner prevented them from attending care [13]. Lack of emotional and instrumental support from family members has also been identified as a barrier to prenatal care for disadvantaged African American women [54].

Similar to our findings, early studies described feeling physically unwell as a deterrent to prenatal care frequently reported by low-income women [13,55]. Recent reviews [25,45] have not addressed feeling unwell as a barrier to prenatal care. While many women experience discomfort in pregnancy, the subset of low-income women with inadequate prenatal care appear to be particularly influenced by their physical symptoms. It may be that, for these women, experiencing pregnancy within the context of other psychosocial, personal, and structural challenges allows the physical challenges to play a larger role in their decisions about seeking or continuing prenatal care.

While lack of transportation and child care are widely recognized as two important barriers to obtaining prenatal care for low-income women in general [25], this study demonstrated that “having transportation problems” and “having child care problems” were distinguishing factors between women who obtained adequate prenatal care and those who did not. Women who identified transportation and child care as problems may be less able than others to resolve these issues, perhaps due to lack of resources or a reduced personal capacity.

### Facilitators

A higher proportion of women with inadequate prenatal care indicated that getting assistance with transportation and child care and receiving financial and emotional support would make “a lot” of difference in helping them get more prenatal care than they did, compared to those with adequate prenatal care. This is consistent with the demographic characteristics of the cases, who had higher proportions of women with a low family income (<$20,000 CAD) and being single, divorced or separated compared to controls. In a meta-synthesis of qualitative studies describing barriers to prenatal care, Downe et al. found that low-income women considered the value of expending their personal resources (e.g., time, money) against the value obtained by attending prenatal care [45].

### Implications for policy and practice

The results of this study point to a need to enhance service delivery by finding ways to make prenatal care more accessible and convenient for inner-city women while minimizing the barriers that study participants identified. For example, providing on-site child care and facilitating transportation may make a significant difference in reducing inadequate prenatal care. Promoting public awareness about the importance of prenatal care and the benefits it holds for the baby’s health may address the attitudinal barrier that prenatal care is not necessary, while building on the value that most often motivates women to seek prenatal care. Programs that emphasize the wellness value of prenatal care and present pregnancy as a normal and healthy life event might help to improve participation by Aboriginal women. Service delivery models such as group prenatal care may provide a sense of personal empowerment to women who prefer to “take care of themselves” and also address a number of other barriers discussed above. In their review of innovative strategies aimed at reducing disparities in the quality of prenatal care, Lu et al. make a number of suggestions in addition to group care, such as enhanced prenatal care (combined with ancillary services) and the use of health information technology to promote prenatal care [56].

Providers of prenatal care should recognize that their behavior towards women, coupled with structural issues such as long wait times and inconvenient clinic hours, have a greater impact on whether women return for follow-up than many may have thought. Caregivers are encouraged to take time to conduct psychosocial assessments and intervene as appropriate to provide support and help reduce stress that may be contributing to a woman’s under-use of available services.

Finally, this study underscores the complex challenges faced by many disadvantaged women in taking advantage of health care services in Canada. Our strong findings on psychosocial and resource-related risk factors for inadequate prenatal care (e.g., family problems, partner abuse, fear of child apprehension, homelessness) argue for an “upstream” approach to reducing disparities in service use. Only by looking at the social, political and environmental contexts in which prenatal care is delivered in various cities across the country will service providers be able to address the full range of barriers that prevent women from using available care.

### Strengths and limitations of the study

This study adhered to the guidelines for case–control studies listed in the Strengthening the Reporting of Observational Studies in Epidemiology (STROBE) statement [57]. Refer to Additional file 3 for STROBE checklist. Strengths of this study included a large sample size, use of a structured questionnaire and interviewer training to reduce potential bias due to lack of blinding to case–control status, and classification of adequacy of prenatal care utilization using an accepted index. The questionnaire was an effective and efficient means of eliciting information about barriers, motivators and facilitators related to use of prenatal care, but lacked established reliability and validity. The potential for recall bias is a limitation of the study, as women were interviewed about their prenatal care during the postpartum period and it may have been difficult for some women to remember facts clearly from the previous nine months. The potential existed for a woman to be interviewed more than once over the three-year period, although, to our knowledge, this occurred only once. Although 93 immigrant women participated in the study, non-English speaking immigrant women may have been under-represented, as we only conducted 9 interviews that required use of an interpreter. The analysis was stratified by neighborhood, but there was no adjustment for other characteristics. The possibility exists that some of the differences in barriers or motivators occurred because of differences in characteristics of the cases and controls. Multiparous women, for example, may be more likely to believe they can take care of themselves during pregnancy, and their use of prenatal care may be affected by their experiences in a previous pregnancy. The provincial Personal Health Information Act prohibited us from collecting information on women who did not agree to be approached by the research nurse to discuss their participation in the study. We were therefore unable to calculate an accurate response rate (because the research nurse could not verify if non-participants met the inclusion criteria), and it is unknown whether the characteristics of women who agreed to participate differed from those who refused to participate. Because the sample was limited to inner-city women, caution needs to be used in generalizing the findings to women living in suburban or rural areas. Finally, case–control studies provide evidence of an association but they do not demonstrate causation.

## Conclusions

Although all women in this study lived in the same group of disadvantaged neighborhoods, several psychosocial, attitudinal, economic and structural barriers and a variety of motivators differentiated those women who received inadequate care from those who received adequate care. This study highlights the heterogeneity among inner-city women with respect to their experiences with prenatal care and their perceptions of factors that help or hinder them in accessing this care. The results can be used to inform the assessment of inner-city women who are at greatest risk for inadequate prenatal care and to design interventions to enhance facilitators and motivators and reduce barriers to care.

## Abbreviations

GINDEX: Graduated index of prenatal care utilization; OR: Odds ratio; CI: Confidence interval; SD: Standard deviation; CAD: Canadian dollar; PNC: Prenatal care; MB: Manitoba; N: Number of participants; %: Percent.

## Competing interests

The authors declare that they have no competing interests.

## Authors’ contributions

MIH wrote the grant application, directed the implementation of the study protocol, and had overall responsibility for the research. MM, LE, WS, MEH, PG, LT, and CC contributed to conception and design of the study, and interpretation of the results. HM coordinated the study. MIH and HM drafted the manuscript. All authors provided feedback on the draft manuscript, and read and approved the final manuscript.

## Pre-publication history

The pre-publication history for this paper can be accessed here:

http://www.biomedcentral.com/1471-2393/14/227/prepub

## Supplementary Material

Additional file 1 **Socio-demographic Characteristics of Winnipeg Neighbourhoods, 2006.** Describes characteristics of each of the 25 neighborhoods in Winnipeg (average family income, percentage of population who are unemployed, reporting Aboriginal status, without a high school diploma, single parent families, immigrants), based on data from the 2006 Canadian Census. Click here for file

Additional file 2 Excerpt of questions related to motivators, barriers and facilitators from the Structured Interview Guide for the study “Factors associated with inadequate prenatal care among inner-city women in Winnipeg,” Principal Investigator: Dr. Maureen Heaman, College of Nursing, Faculty of Health Sciences, University of Manitoba. Click here for file

Additional file 3 **Checklist of Recommendations for Reporting of Case–control Studies Using the STROBE Guidelines.** Indicates where each of the recommended items is reported in the manuscript. Click here for file
