# Supplementary material for: Barriers, motivators and facilitators related to prenatal care utilization among inner-city women in Winnipeg, Canada: a case–control study
Source: BMC Pregnancy Childbirth. 2014 Jul 15;14:227. doi: 10.1186/1471-2393-14-227 (PMC4223395; doi:10.1186/1471-2393-14-227)
Supplement: Additional file 1 — Socio-demographic Characteristics of Winnipeg Neighbourhoods, 2006. Describes characteristics of each of the 25 neighborhoods in Winnipeg (average family income, percentage of population who are unemployed, reporting Aboriginal status, without a high school diploma, single parent families, immigrants), based on data from the 2006 Canadian Census. [file 1471-2393-14-227-S1.docx]

**Additional File 1: Socio-demographic Characteristics of Winnipeg Neighbourhoods, 2006^1^**

| **Neighbourhood** | **Education ^2^** | **Average Family Income ^3^** | **Single Parent ^4^** | **Unemployed ^5^** | **Aboriginal ^6^** | **Recent Immigrants ^7^** |
| --- | --- | --- | --- | --- | --- | --- |
| Assiniboine South | 15.9% | $106,617 | 12.3% | 4.2% | 3.8% | 1.5% |
| Transcona | 26.5% | $64,865 | 18.2% | 5.2% | 9.2% | 1.5% |
| St. James - Assiboine West | 19.6% | $65,935 | 17.4% | 4.4% | 7.6% | 1.3% |
| St. James - Assiboine East | 20.5% | $58,095 | 18.2% | 4.3% | 8.1% | 1.5% |
| Fort Garry North | 15.4% | $88,954 | 10.7% | 4.2% | 3.3% | 3.3% |
| Fort Garry South | 15.6% | $68,316 | 15.1% | 5.5% | 5.8% | 6.7% |
| St. Vital North | 24.0% | $55,058 | 19.6% | 4.9% | 9.6% | 3.9% |
| St. Vital South | 17.1% | $81,211 | 13.2% | 4.0% | 6.2% | 1.9% |
| St. Boniface West | 23.5% | $49,285 | 23.5% | 4.7% | 12.6% | 1.7% |
| St. Boniface East | 19.2% | $82,775 | 13.7% | 3.7% | 7.6% | 1.1% |
| River East - West | 25.8% | $59,037 | 17.3% | 4.7% | 5.6% | 2.4% |
| River East - East | 25.3% | $67,622 | 19.0% | 4.7% | 8.9% | 3.1% |
| **River East – South*** | **33.1%** | **$43,620** | **30.7%** | **5.3%** | **18.7%** | **3.6%** |
| **Seven Oaks West*** | **26.5%** | **$70,704** | **18.7%** | **5.1%** | **8.2%** | **7.5%** |
| Seven Oaks East | 26.5% | $58,706 | 19.4% | 4.9% | 8.5% | 3.8% |
| Inkster West | 23.7% | $72,581 | 16.1% | 4.4% | 8.4% | 6.7% |
| **Inkster East*** | **38.3%** | **$42,036** | **34.5%** | **6.7%** | **25.4%** | **6.3%** |
| **Point Douglas North*** | **34.7%** | **$45,576** | **29.5%** | **6.0%** | **21.1%** | **4.1%** |
| **Point Douglas South*** | **50.2%** | **$30,523** | **47.2%** | **14.3%** | **45.4%** | **4.0%** |
| **Downtown West*** | **27.1%** | **$48,530** | **26.5%** | **5.8%** | **13.7%** | **6.0%** |
| **Downtown East*** | **28.6%** | **$31,338** | **35.8%** | **11.7%** | **21.8%** | **12.0%** |
| River Heights West | 16.2% | $72,785 | 16.9% | 4.6% | 4.6% | 3.0% |
| **River Heights East*** | **15.1%** | **$52,828** | **22.2%** | **4.4%** | **9.7%** | **3.9%** |
| *****The 8 neighbourhoods involved in the study of barriers, motivators and facilitators related to prenatal care utilization among inner-city women in Winnipeg, Canada | | | | | | |
| ^1^ based on data from the 2006 Canadian census | | | | | | |
| ^2^ percentage of population over 15 years of age without a diploma, certificate, or degree | | | | | | |
| ^3^ average household income of census families | | | | | | |
| ^4^ percentage of population of census families headed by a single parent, male or female | | | | | |  |
| ^5^ percentage of population in the labour force over 15 years of age who are not employed in 2006 | | | | | | |
| ^6^ percentage of population reporting Aboriginal status | | | |  |  |  |
| ^7^ percentage of total population immigrated from 2000-2006 | | | | |  |  |
