# Supplementary material for: Barriers, motivators and facilitators related to prenatal care utilization among inner-city women in Winnipeg, Canada: a case–control study
Source: BMC Pregnancy Childbirth. 2014 Jul 15;14:227. doi: 10.1186/1471-2393-14-227 (PMC4223395; doi:10.1186/1471-2393-14-227)
Supplement: Additional file 2 — Excerpt of questions related to motivators, barriers and facilitators from the Structured Interview Guide for the study “Factors associated with inadequate prenatal care among inner-city women in Winnipeg,” Principal Investigator: Dr. Maureen Heaman, College of Nursing, Faculty of Health Sciences, University of Manitoba. [file 1471-2393-14-227-S2.doc]

# Additional File 2: Excerpt of questions related to motivators, barriers and facilitators from the Structured Interview Guide for the study “Factors associated with inadequate prenatal care among inner-city women in Winnipeg,” Principal Investigator: Dr. Maureen Heaman, College of Nursing, Faculty of Health Sciences, University of Manitoba

**Note:** The following questions were adapted with permission from Dr. Allan A. Johnson, PhD, FASAHP, College of Nursing and Allied Health Sciences, Howard University, Washington, DC. Dr. Johnson was the Principal Investigator for a study on barriers, motivators, and facilitators of prenatal care utilization as part of the NIH-DC Initiative to Reduce Infant Mortality in Minority Populations. Refer to the following references for more information:

Johnson AA, El Khorazaty MN, Hatcher BJ, Wingrove BK, Milligan R, Harris C *et al*.: **Determinants of late prenatal care initiation by African American women in Washington, DC.** *Matern Child Health J* 2003, **7**(3):103-114.

Johnson AA, Hatcher BJ, El-Khorazaty MN, Milligan RA, Bhaskar B, Rodan MF *et al*.: **Determinants of inadequate prenatal care utilization by African American women.** *J Health Care Poor Underserved* 2007, **18**(3):620-636.

**Reasons for getting prenatal care (Motivators)**

1. I will now read some reasons why some women get prenatal care. Please tell me whether or not that reason made you go for prenatal care.

*Did you get prenatal care......*

**YES NO N/A**

**(No PNC)**

a) to learn how to

protect your health 1 0 7

b) because you were afraid that you

would have problems during

the pregnancy without

prenatal care 1 0 7

c) to talk to someone

about your pregnancy 1 0 7

d) to learn better

health habits 1 0 7

e) to learn about labor

and delivery 1 0 7

f) to have a healthy baby 1 0 7

g) because your family

wanted you to get prenatal care 1 0 7

h) because your husband or

boyfriend wanted you to get prenatal care 1 0 7

I) because your friends wanted

you to get prenatal care 1 0 7

j) because your health care provider

or social worker wanted you to

get prenatal care 1 0 7

**Barriers to prenatal care**

2. **If Participant Started PNC After 1st Trimester Ask:** The following is a list of things that affect women's decisions to get prenatal care. Please tell me if these reasons may have delayed or caused you difficulties in getting prenatal care.

*Did you delay starting prenatal care or have difficulties in getting prenatal care because....*

**If Participant Started PNC During 1st Trimester Ask:** The following is a list of things that affect women's decisions to get prenatal care. Please tell me if these reasons caused you difficulties in getting prenatal care.

*Did you have difficulties in getting prenatal care because...*

**YES NO N/A**

a) you did not know where you

could get prenatal care 1 0 7

b) you could not get an appointment 1 0 7

c) you had to wait too long to get an

appointment 1 0 7

d) your appointment was cancelled by clinic 1 0 7

e) you didn't like the attitudes

of the staff 1 0 7

f) the hours at the clinic

were not convenient for you 1 0 7

g) you didn't think you could

communicate with the staff 1 0 7

h) you had transportation problems 1 0 7

i) you had child care problems 1 0 7

j) you could not get time off from work 1 0 7

k) you had to wait too long in the waiting

room to see your health care provider 1 0 7

3. **If Participant Started PNC After 1st Trimester Ask:** For many women their beliefs regarding prenatal care keep them from getting prenatal care. Please tell me if these reasons caused you to delay starting prenatal care or not go for prenatal care.

*Did you delay starting prenatal care or not go for prenatal care because ....*

**If Participant Started PNC During 1st Trimester Ask:** For many women their beliefs regarding prenatal care keep them from getting prenatal care. Please tell me if these reasons caused you to avoid going for some of your prenatal care visits.

*Did you avoid going for some of your prenatal care visits or not go for some of your prenatal care visits because…*

**YES NO**

a) you were afraid of or did not

like medical tests and examinations 1 0

b) you do not like needles or taking medicine 1 0

c) generally, you do not like health

care workers 1 0

d) you have been dissatisfied with the

care you received 1 0

e) you go to the emergency room or obstetrical triage

unit when there is a problem with your pregnancy 1 0

f) you did not know you were pregnant 1 0

g) you did not think you needed prenatal care 1 0

h) you can take care of yourself during

pregnancy 1 0

i) you get advice about pregnancy

from family and friends 1 0

j) you did not want to be examined

by a man 1 0

k) you did not want people to know you

were pregnant 1 0

l) the pregnancy was unplanned 1 0

m) you were unhappy about being pregnant 1 0

n) you were thinking of having an abortion 1 0

o) You were worried about the risk of your baby being 1 0

apprehended by CFS.

4. **If Participant Started PNC After 1st Trimester Ask:** For many women stress and personal issues in their lives keep them from getting prenatal care. Please tell me if any of these reasons delayed you or caused you difficulties in getting prenatal care.

*Did you delay starting prenatal care or not go for prenatal care because ....*

**If Participant Started PNC During 1st Trimester Ask:** For many women stress and personal issues in their lives keep them from getting prenatal care. Please tell me if any of these reasons caused you difficulties in getting prenatal care.

*Did you have difficulties in going for prenatal care because...*

**YES NO**

a) you did not feel well 1 0

b) of family problems 1 0

c) of problems with your

husband or boyfriend 1 0

d) you got beat up by your

husband or boyfriend 1 0

e) you had been under stress 1 0

f) you were depressed 1 0

g) you did not feel good

about yourself 1 0

h) of personal problems 1 0

i) you were not thinking straight 1 0

j) you forgot the appointment 1 0

k) you were moving a lot 1 0

l) you were/are homeless 1 0

m) you were afraid of crime

near your home or the clinic 1 0

**Facilitators that would assist with getting prenatal care**

5. Please tell me to what extent the following things would help you get more prenatal care than you did? **(SHOW RESPONSE CARD)**

*How much of a difference would it make if....*

**A LOT SOME A LITTLE NONE NA**

a) you got help with completing

forms. Would you say...... 1 2 3 4 7

b) you got incentives - such

as gifts or money.

Would you say...... 1 2 3 4 7

d) you got rides to

the clinic 1 2 3 4 7

e) you got child care

assistance 1 2 3 4 7

f) you had a home visitor 1 2 3 4 7

g) the clinic had hours

convenient for you 1 2 3 4 7

h) you got a call to follow-up

on missed appointments 1 2 3 4 7

I) the staff were easy

to understand 1 2 3 4 7

j) the staff were from the same

country as you 1 2 3 4 7

k) you had financial support 1 2 3 4 7

l) you had emotional support 1 2 3 4 7
