# Supplementary material for: Barriers, motivators and facilitators related to prenatal care utilization among inner-city women in Winnipeg, Canada: a case–control study
Source: BMC Pregnancy Childbirth. 2014 Jul 15;14:227. doi: 10.1186/1471-2393-14-227 (PMC4223395; doi:10.1186/1471-2393-14-227)
Supplement: Additional file 3 — Checklist of Recommendations for Reporting of Case–control Studies Using the STROBE Guidelines. Indicates where each of the recommended items is reported in the manuscript. [file 1471-2393-14-227-S3.docx]

**Additional File 3: Checklist of Recommendations for Reporting of Case-Control Studies Using the STROBE Guidelines**

|  | Item No | Recommendation | Reported |
| --- | --- | --- | --- |
| **Title and abstract** | 1 | (a) Indicate the study’s design with a commonly used term in the title or the abstract | Title and Abstract |
|  |  | (b) Provide in the abstract an informative and balanced summary of what was done and what was found | Abstract |
| Introduction | | |  |
| Background/rationale | 2 | Explain the scientific background and rationale for the investigation being reported | Background |
| Objectives | 3 | State specific objectives, including any prespecified hypotheses | Background |
| Methods | | |  |
| Study design | 4 | Present key elements of study design early in the paper | Background; Methods – Setting, case definition and subject selection |
| Setting | 5 | Describe the setting, locations, and relevant dates, including periods of recruitment, exposure, follow-up, and data collection | Methods – Setting, case definition and subject selection |
| Participants | 6 | (a) Give the eligibility criteria, and the sources and methods of selection of participants. Describe methods of follow-up | Methods – Setting, case definition and subject selection; Methods - Procedure |
|  |  | (b)For matched studies, give matching criteria and number of exposed and unexposed | Frequency matching described in Methods - Procedure |
| Variables | 7 | Clearly define all outcomes, exposures, predictors, potential confounders, and effect modifiers. Give diagnostic criteria, if applicable | Methods – Data collection; Additional File 2 |
| Data sources/ measurement | 8 | For each variable of interest, give sources of data and details of methods of assessment (measurement). Describe comparability of assessment methods if there is more than one group | Methods – Data collection; Additional File 2 |
| Bias | 9 | Describe any efforts to address potential sources of bias | Methods – Procedure; Discussion – Strengths and limitations of the study |
| Study size | 10 | Explain how the study size was arrived at | Methods – Setting, case definition and subject selection |
| Quantitative variables | 11 | Explain how quantitative variables were handled in the analyses. If applicable, describe which groupings were chosen and why | Methods – Data analysis |
| Statistical methods | 12 | (a) Describe all statistical methods, including those used to control for confounding | Methods – Data analysis |
|  |  | (b) Describe any methods used to examine subgroups and interactions | Not applicable |
|  |  | (c) Explain how missing data were addressed | Results – Sample characteristics |
|  |  | (d) If applicable, explain how loss to follow-up was addressed | Not applicable |
|  |  | (e) Describe any sensitivity analyses | Not applicable |
| Results | | |  |
| Participants | 13 | (a) Report numbers of individuals at each stage of study—e.g. numbers potentially eligible, examined for eligibility, confirmed eligible, included in the study, completing follow-up, and analysed | Results – Sample characteristics; Results – Strength and limitations; Tables 3 and 4 |
|  |  | (b) Give reasons for non-participation at each stage | Not applicable |
|  |  | (c) Consider use of a flow diagram | Not provided (deemed not necessary) |
| Descriptive data | 14 | (a) Give characteristics of study participants (e.g. demographic, clinical, social) and information on exposures and potential confounders | Results – Sample characteristics; Tables 1 and 2 |
|  |  | (b) Indicate number of participants with missing data for each variable of interest | Tables 1 and 2 (footnotes); Tables 3 and 4 (denominator for each item is provided) |
| Outcome data | 15 | Report numbers in each exposure category, or summary measures of exposure | Results; Tables 3 and 4 |
| Main results | 16 | (a) Give unadjusted estimates and, if applicable, confounder-adjusted estimates and their precision (e.g. 95% confidence interval). Make clear which confounders were adjusted for and why they were included | Results; Tables 3 and 4 |
|  |  | (b) Report category boundaries when continuous variables were categorized | Table 1 |
|  |  | (c) If relevant, consider translating estimates of relative risk into absolute risk for a meaningful time period | Not relevant |
| Other analyses | 17 | Report other analyses done—e.g. analyses of subgroups and interactions, and sensitivity analyses | Not applicable |
| Discussion | | |  |
| Key results | 18 | Summarise key results with reference to study objectives | Discussion – Summary of key findings |
| Limitations | 19 | Discuss limitations of the study, taking into account sources of potential bias or imprecision. Discuss both direction and magnitude of any potential bias | Discussion – Strengths and limitations |
| Interpretation | 20 | Give a cautious overall interpretation of results considering objectives, limitations, multiplicity of analyses, results from similar studies, and other relevant evidence | Discussion |
| Generalisability | 21 | Discuss the generalisability (external validity) of the study results | Discussion – Strengths and limitations |
| Other information | | |  |
| Funding | 22 | Give the source of funding and the role of the funders for the present study and, if applicable, for the original study on which the present article is based | Acknowledgements |
